# The effect of TEMPOL pretreatment on postoperative cognitive function, inflammatory response, and oxidative stress in aged rats under sevoflurane anesthesia

**DOI:** 10.1002/iid3.1023

**Published:** 2023-09-28

**Authors:** Tianpin Liu, Tianzi Chen, Jianhua Gong, Changchang You, Bo Zhang, Caiyun Luo, Zhigui Liu, Chun Chen

**Affiliations:** ^1^ Department of Anesthesiology, The First College of Clinical Medical Science China Three Gorges University Yichang Hubei China; ^2^ Department of Anesthesiology Yichang Central People's Hospital Yichang Hubei China; ^3^ Department of Hepatobiliary and Pancreatic Surgery, The First College of Clinical Medical Science China Three Gorges University Yichang Hubei China; ^4^ Department of Hepatobiliary and Pancreatic Surgery Yichang Central People's Hospital Yichang Hubei China; ^5^ Department of Anesthesiology The Affiliated Hospital of Guilin Medical University Guilin Guangxi China

**Keywords:** aged rats, cognitive function, inflammatory response, oxidative stress, sevoflurane anesthesia, TEMPOL pretreatment

## Abstract

**Introduction:**

The heterocyclic compound 4‐hydroxy‐(2,2,6,6‐Tetramethylpiperidin‐1‐yl)oxyl (TEMPOL) has a protective effect on neurological function in brain tissues damaged by ischemia and hypoxia. This study explored the effects of TEMPOL pretreatment on postoperative cognitive function in aged rats under sevoflurane anesthesia, focusing on inflammatory response and oxidative stress.

**Methods:**

Sixty male rats were divided into normal control (C), sevoflurane anesthesia (S), TEMPOL pretreatment (T), and sevoflurane anesthesia + TEMPOL pretreatment (ST) groups (15 per group). Groups T and ST rats received continuous intraperitoneal TEMPOL (100 mg/kg) for 3 days, while groups C and S rats were injected with 0.9% saline. After pretreatment, groups S and ST received 3% sevoflurane anesthesia.

**Results:**

Rats in group S exhibited a longer swimming distance, longer escape latency, lower frequency of platform crossing, and shorter dwell time in the targeted quadrant than those in groups C and T. Rats in group ST exhibited a shorter swimming distance, shorter escape latency, higher frequency of platform crossing, and longer dwell time in the targeted quadrant than those in group S. The expressions of interleukin‐6, tumor necrosis factor‐α, inducible nitric oxide synthase, and Ym1/2 messenger ribonucleic acid were higher in groups S and ST rats than in groups C and T rats and lower in group ST rats than in group S rat (*p* < .05). Superoxide dismutase (SOD), total antioxidant capacity (T‐AOC), and glutathione peroxidase (GSH‐Px) were lower, while malondialdehyde (MDA) was higher in groups S and ST rats than in groups C and T rats (*p* < .05). Group ST showed higher SOD, T‐AOC, and GSH‐Px, and lower MDA than group S (*p* < .05).

**Conclusions:**

TEMPOL pretreatment attenuated postoperative cognitive impairment induced by sevoflurane anesthesia in aged rats. This may be attributed to the downregulation of NR2B‐CREB‐BDNF pathway, reducing the inflammatory response and oxidative stress damage in hippocampal tissue.

## Graphical abstract

Tempol pretreatment can reduce the postoperative cognitive impairment induced by sevoflurane anesthesia in aged rats by minimizing the inflammatory response, oxidative stress and regulating the NR2B‐CREB‐BDNF signaling pathway.

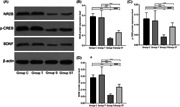

## 1 INTRODUCTION

Postoperative cognitive dysfunction refers to complications of the central nervous system following anesthesia and surgery with impaired memory, confusion, and decline in social skills and comprehension as the main clinical manifestations.^1^, ^2^ Statistics show that the incidence of postoperative cognitive dysfunction at 7 days is as high as 25.8% and is mostly related to age, type of surgery, anesthetic drugs, inhalation concentration, and extracorporeal circulation. Use of general anesthetics is believed to be the main risk factors for inducing cognitive dysfunction after anesthesia.^3^, ^4^, ^5^ General anesthetics can lead to apoptosis and degeneration of neurons in the hippocampi of experimental animals, impairing their memory and spatial learning ability; however, the exact mechanism has not been fully elucidated.^6^ Sevoflurane is a new type of volatile inhalational anesthetic widely used in the induction and maintenance of general anesthesia owing to its rapid metabolism, low stimulation, and low impact on the respiratory and circulatory systems.^7^, ^8^ Similar to other inhaled anesthetics such as isoflurane, sevoflurane can cause cognitive impairment mostly related to the inhibition of neural function in the dentate gyrus of the hippocampus and the induction of neuronal damage resulting from lipid peroxidation.^9^

The heterocyclic compound 4‐hydroxy‐(2,2,6,6‐Tetramethylpiperidin‐1‐yl)oxyl (TEMPOL) is a superoxide dismutase (SOD)‐mimetic agent that inhibits hydroxide production, scavenges oxygen, and has the advantages of membrane permeability, stability, and low molecular weight.^10^ As a novel free radical scavenger, TEMPOL can remove free radicals, inhibit lipid peroxidation, and suppress oxidative damage in nerve cells. TEMPOL pretreatment can change the oxidative environment of brain tissue and has a protective effect on the recovery of neurological function of brain tissue damaged by ischemia and hypoxia.^11^ In recent years, the role of TEMPOL in scavenging free radicals and reducing damage to neural tissue has received considerable attention as it can inhibit lipid peroxidation, suppress inflammatory response, repair damaged synapses, and reduce oxidative damage in brain neuronal cells and vascular endothelial cells.^12^ However, only a few studies have reported the mechanism behind the effects of TEMPOL pretreatment on cognitive function after anesthesia. In this study, we established a rat model of sevoflurane anesthesia and analyzed the protective effects of TEMPOL pretreatment on the postoperative cognitive functions of aged rats under this model and the relevant underlying mechanisms to provide a basis for optimizing anesthetic protocols in the elderly population.

## 2 METHODS

### 2.1 Animal grouping

The experimental procedures conformed to the relevant requirements of the Regulations of the People's Republic of China on the Administration of Laboratory Animals. Sixty aged male Sprague–Dawley rats weighing 500–600 g (mean weight: 556.35 ± 20.15 g; 20–24 months old) were provided by Beijing Viton Lihua Company (animal use license no. XC‐XK [Beijing] 2012‐0003). The rats were divided into four groups using the random number table method: normal controls (group C), sevoflurane anesthesia (group S), TEMPOL pretreatment (group T), and sevoflurane anesthesia + TEMPOL pretreatment (group ST), with 15 rats in each group. All procedures were approved by the Animal Care and Use Committee of the Yichang Central People's Hospital.

### 2.2 Animal experiments

#### 2.2.1 TEMPOLpretreatment and sevoflurane anesthesia

The dosage of TEMPOL was determined according to a literature report^13^ and the results of the preliminary experiment. Rats in groups T and ST were intraperitoneally administered 100 mg/kg TEMPOL (MedChemExpress LLC) for 3 days. Rats in groups C and S were intraperitoneally administered 0.9% saline (Shanghai Xuanya Biotechnology Co. Ltd.). After pretreatment, the rats in groups S and ST were placed in a 50 × 30 × 30 cm anesthesia box within a 37°C constant temperature water bath. Sodium lime was placed at the bottom of the box, and the upper vent of the anesthesia box was connected to the anesthetic to introduce sevoflurane (Emmett Technology Co. Ltd.). The oxygen concentration and flow rate were 50% and 2 L/min, respectively. The lower vent was on the opposite side and connected to an anesthetic gas monitor (Vamos Plus; Dräger Medical). The minimum alveolar concentration (MAC) value was kept at 2.0 MAC. Rats in groups S and ST were administered 3% sevoflurane anesthesia via continuous inhalation for 5 h.^13^ Rats in groups C and T were not administered sevoflurane anesthesia but were placed in an anesthesia box.

#### 2.2.2 Morris water maze

After the administration of final anesthesia, the Morris water maze system (Nanjing Calvin Biotechnology Co., Ltd.) was used to measure the cognitive function of the rats (circular pool, 150 cm diameter × 70 cm height). The escape platform was 12 cm in diameter and 30 cm in height. Skimmed milk powder was used as a shielding agent. A camera was placed 1.5 m above the center of the pool and used to automatically film the swimming path. The water maze experiment was conducted at constant water temperature of 20–22°C for 7 days in a quiet environment. Place navigation was analyzed on Days 1–6, with an interval of 15 min, four times/day, with the help of two training sessions each in the morning and afternoon. The rats were observed from entry into the water until they found the platform (escape latency); when the time to find the platform exceeded 60 s, they were guided to find the platform, and it was recorded as 60 s. When the rats reached the platform on their own, they were allowed to rest for 30 s, and the average value of four measurements was taken as the final value. On Day 7, the spatial probe test was performed after removing the platform. Rats entered the water at the midpoint of quadrant 1 and swam for 120 s. The number of times the rats crossed the platform and the time they spent in the quadrant where the platform was located were recorded using video cameras. On the day after the water maze experiment, the rats were anesthetized by intraperitoneal injection of 2% pentobarbital sodium (0.3 mL/100 g body weight). The brain tissue was rinsed by perfusing with it saline, immediately removed, and placed on ice. The hippocampal tissue was separated and stored in liquid nitrogen and then stored in an HD‐86L830 –80°C ultralow temperature refrigerator (Nanjing Beden Medical Co., Ltd.).

#### 2.2.3 Blood gas analysis

Five rats from each group were selected for this study. After drug administration, the rats were anesthetized by intraperitoneal injection with 2% pentobarbital sodium (0.3 ml/100 g body weight). A 1 mL heparinized syringe was used to puncture the left ventricle and draw the arterial blood samples. During anesthesia, the pH, partial pressure of oxygen (PO_2_), partial pressure of carbon dioxide (PCO_2_), and blood oxygen saturation (SO_2_) were measured using a blood gas analyzer (Bayer).

#### 2.2.4 Detection of SOD, total antioxidant capacity (T‐AOC), malondialdehyde (MDA), and glutathione peroxidase (GSH‐Px) using the enzyme‐linked immunosorbent assay (ELISA) method

Hippocampal tissues from five rats of each group were used in this experiment. Sodium chloride solution (0.9%) was added nine times to prepare the tissue homogenate, which was centrifuged using a tabletop high‐speed centrifuge M1324 (Shanghai Fuze Trading Co., Ltd.). Supernatants were obtained, and the levels of SOD, T‐AOC, MDA, and GSH‐Px were measured using the ELISA method (Shanghai Lianmai Biological Engineering Co.).

#### 2.2.5 Detection of interleukin‐6 (IL‐6), tumor necrosis factor‐α (TNF‐α), Ym1/2, and inducible nitric oxide synthase (iNOS) in hippocampal tissue using a real‐time quantitative polymerase chain reaction (PCR) method

Hippocampal tissues from five rats of each group were used in this experiment. Total ribonucleic acid (RNA) was extracted from the hippocampal tissues using the TRIzol method (Wuhan Purity Biotechnology Co., Ltd.), diluted, and reverse‐transcribed to copy deoxyribonucleic acid (cDNA). Primers were designed using the Primer5 software (primers were synthesized by Shanghai Bioengineering Company), including IL‐6: upstream: TGCAAGAGACTTCCATCCAGTT, downstream: GAAGTAGGGAAGGCCGTGG; TNF‐α: upstream: GCACCACCATCAAGGACTC, downstream: TGAGACAGAGGCAACCTGAC; Ym1/2: upstream: CAGGGTAATGAGTGGGTTGG, downstream: CACGGCACCTCCTAAATTGT; and iNOS: upstream: GGCAGCCTGTGAGACCTTTG, downstream: GCATTGGAAGTGAAGCGTTTC. The PCR amplification conditions were 95°C for 3 min, 95°C for 30 s, 55°C for 30 s, 72°C for 50 s (35 cycles), 72°C for 8 min, and stored at 4°C. The relative expression of IL‐6, TNF‐α, iNOS, and Ym1/2 was calculated using $2^{‐∆∆C_{t}}$ with actin as an internal reference.

#### 2.2.6 Western blot

Hippocampal tissues from five rats of each group were used in this experiment. Total proteins in the hippocampal tissues were extracted with RIPA lysate (Thermo Fisher Scientific). Protein quantification was performed by bicinchoninic acid method, SDS‐polypropylene gel electrophoresis, membrane transfer, skim milk powder sealing followed by treatment with primary antibodies (N‐methyl‐d‐aspartate receptor subunit 2B [NR2B], p‐cAMP responsive element binding protein [p‐CREB], brain‐derived neurotrophic factor [BDNF], heme oxygenase‐1 [HO‐1], nuclear factor‐E2 related factor 2 [Nrf2], glyceraldehyde‐3‐phosphate dehydrogenase [GAPDH] antibodies; 1:1000) and incubation at 4°C for 30 min and then overnight. The membrane was washed thrice with TBST buffer for 5 min, incubated for 40 min with horseradish peroxidase‐labeled secondary antibody (1:1000), washed thrice with TBST buffer, and developed using electrochemiluminescence. GAPDH was used as an internal reference, and the grayscale values of NR2B, p‐CREB, BDNF, HO‐1, and Nrf2 protein bands were calculated using ImageJ (National Institutes of Health) software.

#### 2.2.7 Statistical analysis

The sample size of the experimental rats was estimated using a free online software (www.powerandsamplesize.com). The sample size of each group was determined to be 15 rats based on reports in the literature and the actual experimental expenses of this study. Statistical Package for the Social Sciences (SPSS) software (version 24.0; IBM Corp.) and measurement data conforming to normal distribution were expressed as $\bar{x}\pm s$, and one‐way analysis of variance was used for comparison between multiple groups, followed by post hoc least significance difference (LSD) *t* test, with *p* < .05 indicating statistical significance.

## 3 RESULTS

### 3.1 Effect of TEMPOL pretreatment on postoperative blood gas analysis

The differences in pH, PO_2_, PCO_2_, and SO_2_ between the four groups were not statistically significant (*p* > .05), and none of the rats in the four groups exhibited hypoxia, respiratory depression, uniform respiration, or red lips during anesthesia (Table 1).

**Table 1:** Comparison of blood gas analysis (*χ* ± S).

Group	Number of cases	pH	PO_2_ (mmHg)	PCO_2_ (mmHg)	SO_2_ (%)
C	15	7.42 ± 0.06	234.65 ± 22.02	38.52 ± 3.94	95.69 ± 2.91
T	15	7.39 ± 0.08	229.98 ± 23.16	37.06 ± 4.03	96.13 ± 4.34
S	15	7.41 ± 0.08	231.95 ± 22.75	38.96 ± 4.16	95.83 ± 3.22
ST	15	7.45 ± 0.11	235.64 ± 23.76	39.03 ± 4.25	96.09 ± 4.09

Abbreviations: PCO_2_, partial pressure of carbon dioxide; PO_2_, partial pressure of oxygen; SO_2_, blood oxygen saturation.

### 3.2 Sevoflurane caused cognitive dysfunction in aged rats

**Figure 1:**
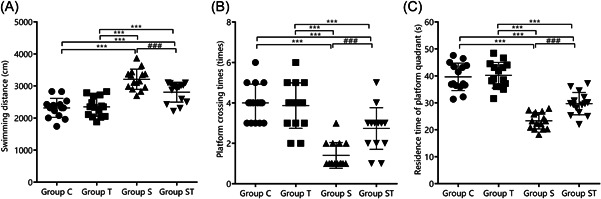
Comparison of behavioral indicators of water maze in each group (*n* = 15). TEMPOL pretreatment improves water maze behavior and alleviated cognitive impairment in aged rats under sevoflurane anesthesia: (A) swimming distance, (B) number of platform crossings, and (C) dwell time in the targeted quadrant. Compared with groups C and T, ***p < .001; compared with group S, ^###^ *p* < .001. A one‐way analysis of variance was used to compare the swimming distance, number of platform crossings, and dwell time in the targeted quadrant among the groups; the differences were statistically significant (*p* < .001).

The differences in swimming distance, number of platform crossings, and dwell time in the targeted quadrant were not statistically significant between groups C and T (*p* > .05). Rats in group S had a longer swimming distance, lower number of platform crossings, and shorter dwell time at the targeted quadrant than those in groups C and T (*p* < .05). Rats in group ST had a shorter swimming distance, higher number of platform crossings, and longer dwell time in the targeted quadrant than those in group S (*p* < .05). This indicates that TEMPOL pretreatment improved water maze behavior and alleviated impaired cognitive function in aged rats following sevoflurane anesthesia (Figure 1).

### 3.3 Effect of TEMPOL pretreatment on the escape latency at different time points

**Figure 2:**
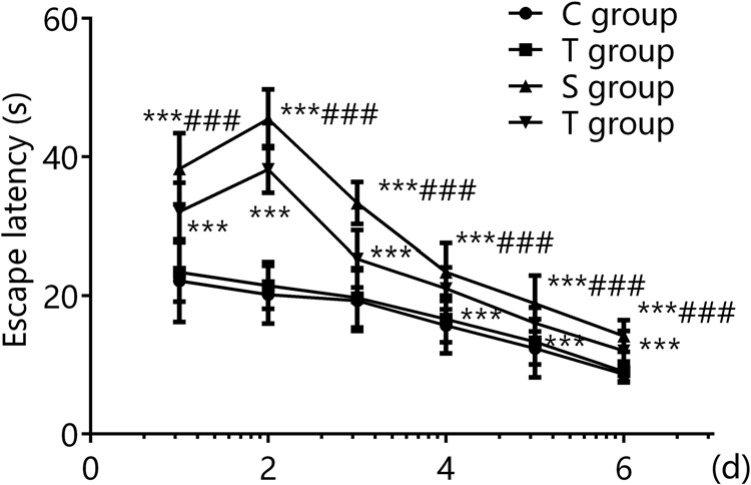
Comparison of escape latency at different time points (*n* = 15). TEMPOL pretreatment improves the escape latency in aged rats under sevoflurane anesthesia and enhance cognitive function. Compared with groups C and T, ***p < .001; compared with group S, ^###^ *p* < .001. A two‐way analysis of variance was used to compare the escape latency at different time points among the groups, and the differences were statistically significant (*p* < .001). There was an interaction between different treatment groups and different time points.

The difference between different time points of escape latency in groups C and T was not statistically significant (*p* > .05). The escape latencies at different time points in groups S and ST were higher than those in groups C and T (*p* < .05), and the escape latencies at different time points in group ST were lower than those in group S (*p* < .05), indicating that TEMPOL pretreatment shortened the escape latency and improved cognitive function in aged rats under sevoflurane anesthesia (Figure 2).

### 3.4 Effect of TEMPOL pretreatment on postoperative inflammatory response

**Figure 3:**
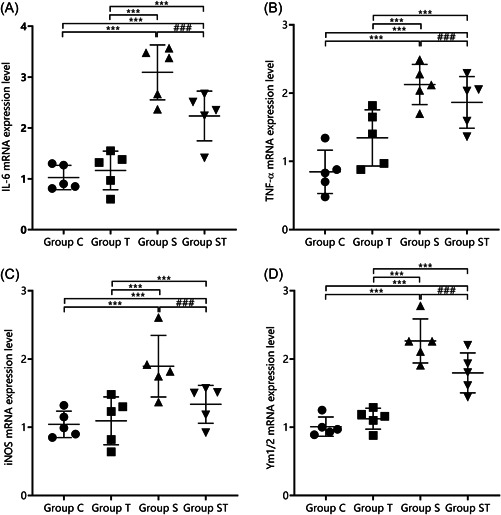
Comparison of mRNA expression of M1 and M2 microglia markers in the hippocampal region of rats (*n* = 3). TEMPOL pretreatment reduces the levels of inflammatory response indicators in M1 microglia in the hippocampal region of aged rats under sevoflurane anesthesia: (A) IL‐6, (B) TNF‐α, (C) iNOS, and (D) Ym1/2. Compared with groups C and T, **p < .01, ***p < .001; compared with group S, ^##^ *p* < .01, ^###^ *p* < .001. A one‐way analysis of variance was used to compare IL‐6, TNF‐α, iNOS, and Ym1/2 among groups, and the difference was statistically significant (*p* < .001). GSH‐Px, glutathione peroxidase; IL, interleukin; iNOS, inducible nitric oxide synthase; MDA, malondialdehyde; SOD, superoxide dismutase; T‐AOC, total antioxidant capacity; TNF, tumor necrosis factor.

The differences in IL‐6, TNF‐α, iNOS, and Ym1/2 mRNA expression in groups C and T were not statistically significant (*p* > .05). IL‐6, TNF‐α, iNOS, and Ym1/2 mRNA expressions were higher in groups S and ST than in groups C and T (*p* < .05) and lower in group ST than in group S (*p* < .05), indicating that TEMPOL pretreatment reduced the inflammatory response of M1 microglia in the hippocampal region of aged rats under sevoflurane anesthesia (Figure 3).

### 3.5 Effect of TEMPOL pretreatment on postoperative oxidative stress levels in aged rats under sevoflurane anesthesia

**Figure 4:**
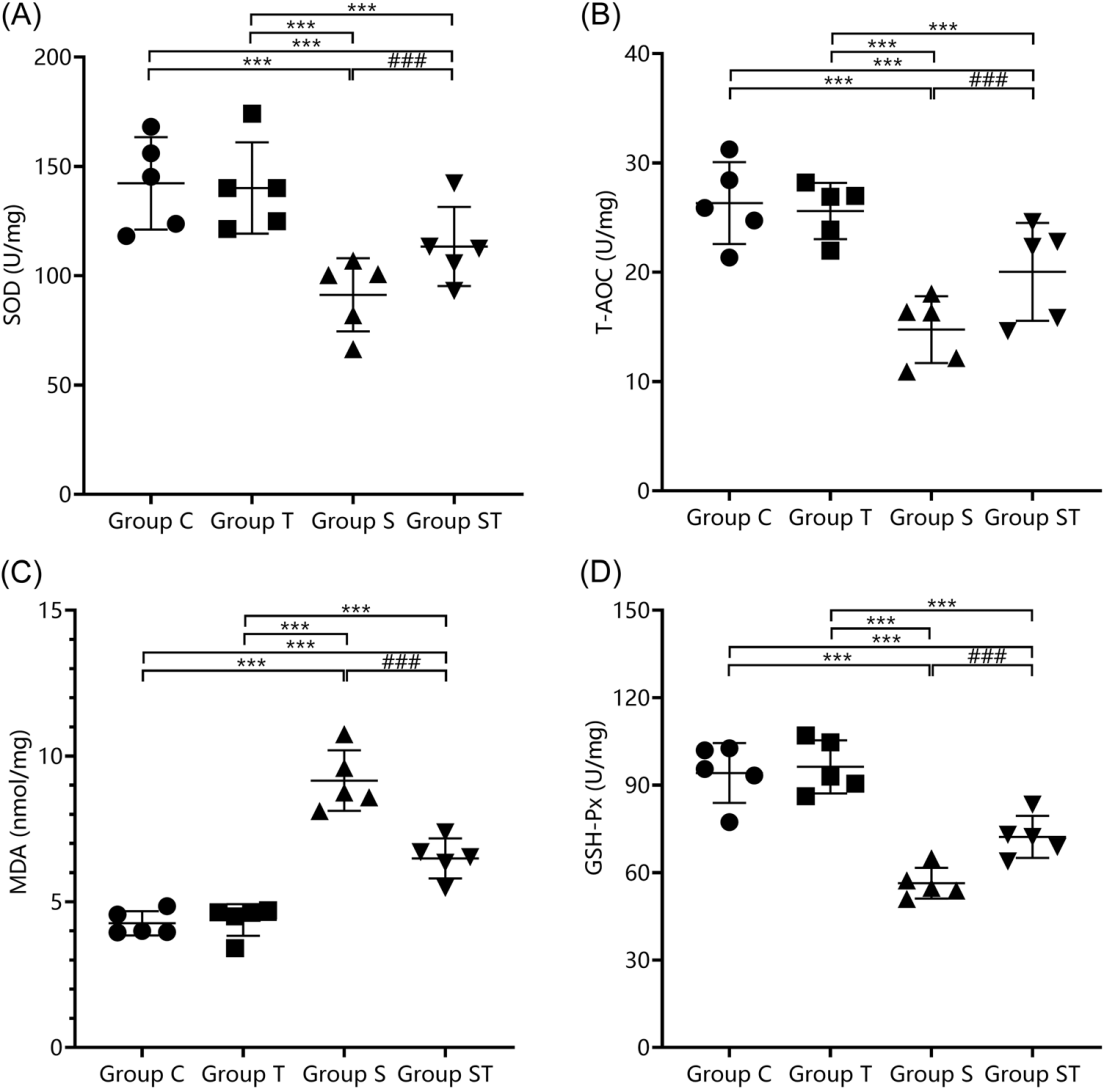
Comparison of oxidative stress indicators in the hippocampal region of rats in each group (*χ* ± S) (*n* = 5). TEMPOL pretreatment reduction of the levels of the oxidative stress indicators: (A) SOD, (B) T‐AOC, (C) MDA, and (D) GSH‐Px in M1 microglia in the hippocampal region of aged rats under sevoflurane anesthesia. Compared with groups C and T, ***p < .001; compared with group S, ^###^ *p* < .001. A one‐way analysis of variance was used to compare SOD, T‐AOC, MDA, and GSH‐Px among groups, and the difference was statistically significant (*p* < .001). GSH‐Px, glutathione peroxidase; MDA, malondialdehyde; SOD, superoxide dismutase; T‐AOC, total antioxidant capacity.

The differences in SOD, T‐AOC, MDA, and GSH‐Px levels in groups C and T were not statistically significant (*p* > .05). SOD, T‐AOC, and GSH‐Px levels were lower in groups S and ST than in groups C and T, and MDA was higher in groups S and ST than in groups C and T (*p* < .05). SOD, T‐AOC, and GSH‐Px levels were higher in group ST than in group S, and MDA was lower in group ST than in group S (*p* < .05). The results indicate that TEMPOL pretreatment attenuated oxidative stress in aged rats under sevoflurane anesthesia (Figure 4).

### 3.6 Effect of TEMPOL pretreatment on the postoperative NR2B‐CREB‐BDNF signaling pathway

**Figure 5:**
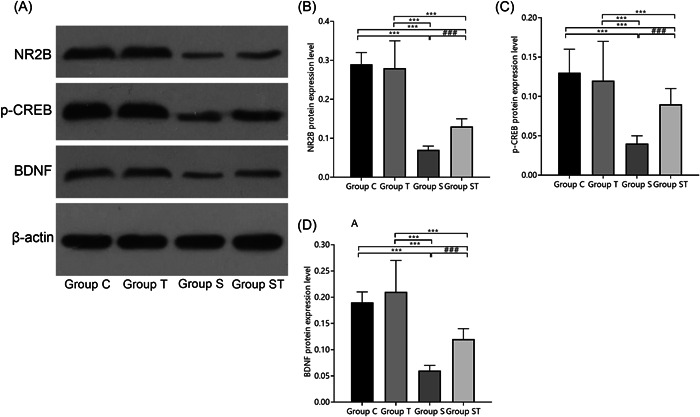
Comparison of NR2B‐CREB‐BDNF signaling pathway protein expression in hippocampal tissue of rats (*n* = 3). TEMPOL pretreatment reduces the expression of NR2B, p‐CREB, and BDNF proteins in M1 microglia in the hippocampal region of aged rats under sevoflurane anesthesia: (A) Western blot image, (B) NR2B, (C) p‐CREB, and (D) BDNF. Compared with groups C and T, ***p < .001; compared with group S, ^###^ *p* < .001. A one‐way analysis of variance was used to compare NR2B, p‐CREB, and BDNF among groups, and the difference was statistically significant (*p* < .001). BDNF, brain‐derived neurotrophic factor; N2RB, N‐methyl‐d‐aspartate receptor subunit 2B; p‐CREB, p‐cAMP responsive element binding protein.

The differences in NR2B, p‐CREB, and BDNF protein expression in groups C and T were not statistically significant (*p* > .05). NR2B, p‐CREB, and BDNF protein expression was lower in groups S and ST than in groups C and T (*p* < .05) and were higher in group ST than in group S (*p* < .05), indicating that TEMPOL pretreatment may attenuate cognitive decompensation in aged rats under sevoflurane anesthesia by modulating the NR2B‐CREB‐BDNF signaling pathway (Figure 5).

### 3.7 Effect of TEMPOL pretreatment on the expression of HO‐1 and Nrf2 proteins in postoperative hippocampal tissues

**Figure 6:**
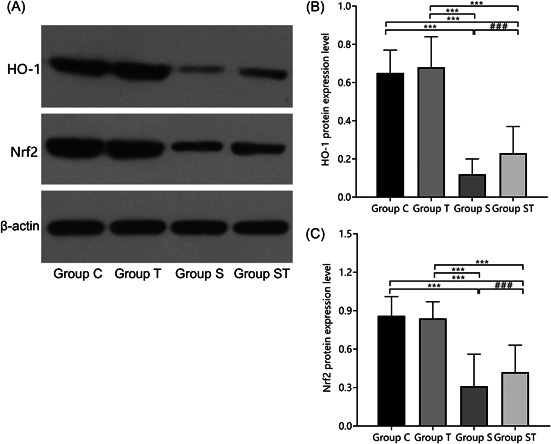
Comparison of HO‐1 and Nrf2 protein expression in hippocampal tissue of rats (*n* = 3). TEMPOL pretreatment increases HO‐1 and Nrf2 protein expression in M1 microglia in the hippocampal region of aged rats under sevoflurane anesthesia: (A) Western blot image, (B) HO‐1, and (C) Nrf2. Compared with groups C and T, ***p < .001; Compared with group S, ^###^ *p* < .001. A one‐way analysis of variance was used to compare HO‐1 and Nrf2 among groups, and the difference was statistically significant (*p* < .001). HO‐1, heme oxygenase‐1; Nrf2, nuclear factor‐E2 related factor 2.

The differences in HO‐1 and Nrf2 protein expression in groups C and T were not statistically significant (*p* > .05). HO‐1 and Nrf2 protein expression was lower in groups S and ST than in groups C and T (*p* < .05). HO‐1 and Nrf2 protein expression was higher in group ST than in group S (*p* < .05). The results indicated that TEMPOL pretreatment may reduce oxidative stress injury in aged rats under sevoflurane anesthesia by upregulating HO‐1 and Nrf2 protein (Figure 6).

## 4 DISCUSSION

Sevoflurane is mostly used for intraoperative anesthesia, and although the surgical procedure ITSELF may impact neuroinflammation, sevoflurane can exacerbate this neural damage. Sevoflurane can induce and exacerbate neuroinflammation by stimulating the production of inflammatory factors such as IL‐1β and TNF‐α; it can also induce neuronal oxidative damage by activating calcium‐dependent proteases, leading to the generation of a large number of oxygen free radicals, which promotes neuronal apoptosis and results in cognitive dysfunction.^14^, ^15^, ^16^ TEMPOL is a novel free radical scavenger with the advantages of high stability and low molecular weight and has been widely used in research regarding ischemia‐reperfusion in the kidney, liver, and spinal cord.^17^ Qi et al.^18^ found via in vitro experiments of Takotsubo syndrome that TEMPOL could improve oxidative dysfunction of mitochondria through the reactive oxygen/mitochondrial/antiapoptotic/p38 MAPK pathways. In the present study, pretreatment with TEMPOL significantly ameliorated the severe cognitive impairment in rats after sevoflurane anesthesia.

The pH, PO_2_, and PCO_2_ of the rats in all groups during anesthesia were within the normal range, and there were no signs of hypoxia or respiratory depression, which excluded the influence of physiological factors on the accuracy of the subsequent study. The swimming distances and escape latencies at different time points were longer in groups S and ST than in groups C and T, and the number of crossings and dwell times in the targeted quadrant were lower and shorter than those in groups C and T, indicating that the rats showed severe cognitive impairment after sevoflurane administration. These results are consistent with the those of previous studies.

In the present study, rats in group ST had a shorter swimming distance, lower escape latency at different time points, higher number of platform crossings, longer time spent in the targeted quadrant, and higher levels of NR2B, p‐CREB, and BDNF protein than those in group S, indicating that TEMPOL pretreatment attenuated cognitive decompensation in aged rats under sevoflurane anesthesia, and its mechanism may be related to the regulation of the NR2B‐CREB‐BDNF signaling pathway.

Anesthesia can enhance central inflammatory sensitivity, causing the release of inflammatory factors such as IL‐6 and TNF‐α.^19^ Additionally, the neuroinflammatory response can affect hippocampus‐dependent memory retention and immune regulation of the central nervous system, leading to impaired cognitive function and forming a vicious cycle.^20^ Moreover, the balance between oxidation and antioxidation can be disturbed, thus generating excessive clusters of reactive oxygen species, aggravating mitochondrial dysfunction, brain homeostatic imbalance, and oxidative stress‐related injury, which can cause abnormal structure of hippocampal tissue and induce cognitive dysfunction.^21^, ^22^ Nrf2 is a key transcription factor for cellular redox homeostasis and can enter the nucleus to bind to antioxidant response elements (AREs), which initiate the regulation of downstream anti‐inflammatory factors, antioxidant proteins, immune factors, and many other target genes.^23^, ^24^ HO‐1 catalyzes the release of biliverdin through heme catabolism in the presence of oxygen and coenzyme II, thereby inhibiting lipid peroxidation.^25^ Zhu^26^ found that knockdown of the Nrf2 gene in mice increased the levels of reactive oxygen species, decreased HO‐1 activity, and reduced antioxidative stress effects, while Wang^27^ reported that Nrf2 upregulation in the HO‐1 pathway expression in mouse hippocampal tissue reduced the extent of oxidative stress injury. Another study confirmed that TEMPOL inhibits oxidative stress and attenuates inflammatory responses in the lung tissue by activating the Nrf2/HO‐1 pathway.^28^ The present study's results show that IL‐6, TNF‐α, iNOS, and Ym1/2 mRNA expression and MDA levels were higher in groups S and ST than in groups C and T, and HO‐1 and Nrf2 protein expression, SOD, T‐AOC, and GSH‐Px levels were lower than in groups C and T, indicating that sevoflurane could aggravate the inflammatory response and oxidative stress injury and affect cognitive function. After the exogenous administration of TEMPOL pretreatment, IL‐6, TNF‐α, iNOS, and Ym1/2 mRNA expression and MDA levels were lower, while HO‐1 and Nrf2 protein expression, SOD, T‐AOC, and GSH‐Px levels were higher in group ST than in group S, indicating that rats initiated endogenous protective mechanisms in response to exogenous oxidative stress. Nrf2 protein nuclear translocation further promotes the expression and transcription of the downstream antioxidant gene HO‐1, whereas TEMPOL pretreatment may enhance the anti‐inflammatory response and resistance of hippocampal tissue to oxidative stress by upregulating the Nrf2/HO‐1 pathway in rat hippocampal tissue, similar to the findings of the above study.

The present study had limitations due to the study period and experimental conditions. Cognitive function was not observed in the rats in the immediate and later postoperative periods. Furthermore, this is the first study to consider that changes in the secretion of various hormones in aged female rats can affect the experimental results, we ignored the bias of the experiment, which is a limitation. In a subsequent study, female rats should be included to obtain more comprehensive experimental results. Therefore, the specific mechanism underlying the protective effect of TEMPOL on cognitive function needs to be further explored.

Oxidative stress, caused by an imbalance between the production of reactive oxygen species (free radicals) and antioxidant defense, can lead to tissue damage and various diseases, such as those related to the nervous system. Nrf2 is a transcription factor that plays a critical role in oxidative stress response. Under steady‐state conditions, Kelch‐like ECH‐associated protein 1 (KEAP1) binds to Nrf2 and retains it in the cytoplasm for ubiquitin‐mediated degradation, maintaining a lower level of Nrf2. After oxidative stress, KEAP1 releases Nrf2, which then translocates the activator to the cell nucleus and binds to ARE genes, leading to the expression of cytoprotective genes such as GSH, HO‐1, NADP(H), and NQO1. Therefore, the Nrf2 level is low in normal cells, and the expression only increases after its activation. Only the total Nrf2 level was detected in this study, but this may not be sufficient to illustrate the issue, which is a shortcoming. In subsequent research, additional experiments will be conducted to detect the Nrf2 level in the cell nucleus to illustrate Nrf2 activation further.

This study had another shortcoming. Many oxidants can alleviate the damage to cognitive function caused by anesthesia through the antioxidant stress response. This study mainly observed the anti‐inflammatory and antioxidative stress effects of the novel antioxidant TEMPOL under sevoflurane treatment conditions and did not compare them with those of other antioxidants. TEMPOL, a novel free radical scavenger, is a SOD mimetic with small molecular weight, high stability, and good cell membrane permeability. It has a protective effect on ischemia/reperfusion injury in the spinal cord, liver, and kidneys and can scavenge free radicals, inhibit lipid peroxidation, and inhibit oxidative damage in nerve cells. The structure and function of TEMPOL have already demonstrated its superiority over other antioxidants; hence, no comparison was conducted in this experiment. This is a limitation of the study, and a comparison between different antioxidants will be conducted in further research to demonstrate the superiority of TEMPOL.

In conclusion, TEMPOL pretreatment attenuated postoperative cognitive impairment induced by sevoflurane anesthesia in aged rats. The mechanism may be related to the downregulation of the NR2B‐CREB‐BDNF signaling pathway, which may play a role in reducing the inflammatory response and oxidative stress damage in the hippocampal tissues.

## AUTHOR CONTRIBUTIONS

**Tianpin Liu**: Conceptualization; data curation; formal analysis. **Tianzi Chen**: Conceptualization; data curation; formal analysis. **Jianhua Gong**: Formal analysis; investigation. **Changchang You**: Methodology; validation. **Bo Zhang**: Investigation; validation. **Caiyun Luo**: Data curation; methodology. **Zhigui Liu**: Conceptualization; formal analysis; funding acquisition; writing—original draft; writing—review and editing. **Chun Chen**: Conceptualization; formal analysis; writing—original draft; writing—review and editing. All authors have read and approved the final manuscript.

## CONFLICT OF INTEREST STATEMENT

The authors declare no conflict of interest.

## ETHICS STATEMENT

All procedures were approved by the Animal Care and Use Committee of the Yichang Central People's Hospital.

## Data Availability

All data generated or used during the study are present in the submitted article.
